# Correction

**Published:** 1875-10

**Authors:** J. G. Westmoreland


CORRECTION.—In the announcement of the insuing course of lectures in Atlanta Medical College, as well as in the advertisement in this JOURNAL, the name of Professor A. W. Griggs was inadvertently left out. Dr. Griggs holds the same connection with the college as heretofore.

---

INFIRMARY for the treatment of FEMALE DISEASES at the Air-Line House, Pryor Street, Atlanta, Ga., by

J. G. WESTMORELAND, M.D.

